# Clinical and Echocardiographic Profile of Congenital Heart Diseases in the 0-12-Year Age Group in a Tertiary Care Medical Institute in Eastern India: A Retrospective, Cross-Sectional Study

**DOI:** 10.7759/cureus.26114

**Published:** 2022-06-20

**Authors:** Sunil Kishore, Manish Kumar, Amit Kumar, Anand Gupta, Chandrabhanu Chandan, Anshuman Anshuman, Jayant Prakash, Shalini Sinha, Neeraj Kumar

**Affiliations:** 1 Pediatrics, Indira Gandhi Institute of Medical Sciences, Patna, IND; 2 Cardiology, Indira Gandhi Institute of Medical Sciences, Patna, IND

**Keywords:** tetralogy of fallot, ventricular septal defect, congenital heart disease, congestive heart failure, echocardiography

## Abstract

Background

This study aimed to determine the clinical and echocardiography profile of congenital heart diseases (CHDs) among admitted children as well as patients presenting to the outpatient department of the Indira Gandhi Institute of Medical Sciences, Patna, which is a tertiary care and apex institute located in Bihar, India.

Methodology

A retrospective, cross-sectional study was conducted in a tertiary care hospital from January 2019 to January 2021. In total, 200 patients aged 0-12 were enrolled in the study. The study design was exploratory, with a proforma drafted to study patients with features suggestive of CHDs. All pediatric echocardiography was performed by a trained cardiologist. Records were obtained from the departmental record-keeping register, and reports were available for analyzing the data. For data collection, cardiology and pediatric department registers were reviewed and all CHD data were collected. Data analysis was done using SPSS software version 25.0 (IBM Corp., Armonk, NY, USA).

Results

Of the 200 children with CHDs, 142 were diagnosed to have acyanotic heart disease (ACHD), while 58 had cyanotic congenital heart disease (CCHD). Among 200 cases of CHD, ventricular septal defect (VSD) constituted 62 cases comprising 31% of the total CHD cases and 44% of all ACHD cases. Atrial septal defect (ASD) was the second most common CHD comprising 23% of all CHD cases. Tetralogy of Fallot (TOF) constituted 23 cases accounting for 11.5% of all CHD cases. It was the most common CCHD. Based on the age at the time of presentation, 90 (45%) cases were diagnosed below one year of age. Congestive heart failure (CHF) was most common in ACHD comprising 30% compared to CCHD in which only 7% of cases had CHF. This finding was statistically significant (p < 0.05). Analyzing the symptoms of different CHDs, of both ACHDs and CCHDs, the common symptoms were fast breathing (38%).

Conclusions

Among ACHD patients, 31% VSD and 21% ASD were noted. In CCHD, TOF was the most common with 11.5% of cases. Respiratory tract conditions were the most common comorbidities encountered. Because this is one of the first studies conducted in Bihar in the pediatric age group, it can help know the prevalence of CHDs in this region and will be useful for developing policies by stakeholders.

## Introduction

Congenital heart defect (CHD) is defined as an anatomic malformation of the heart or great vessels that occurs during intrauterine development, irrespective of the age at presentation and represents a major global health problem [1]. It remains the leading cause of death in children with malformations [2]. It is the most common congenital birth defect affecting 28% of all major congenital anomalies [1]. The global prevalence of CHD is eight per 1,000 live births [2]. In India, different studies have reported a prevalence between five and eleven per 1,000 live births [3-5]. CHDs are categorized as acyanotic congenital heart disease (ACHD) and cyanotic congenital heart disease (CCHD) according to the pathophysiology and the affected heart structure [6]. The acyanotic lesion consists of septal cardiac defects such as atrial septal defect (ASD), ventricular septal defect (VSD), and atrioventricular canal defects (AVSD). It also includes left ventricular outflow obstructive lesions such as aortic stenosis (AS) and coarctation of the aorta (COA). CCHD includes tetralogy of Fallot (TOF), transposition of great arteries (TGA), total anomalous pulmonary venous returns (TAPVC), hypoplastic left heart syndrome (HLHS), truncus arteriosus, and tricuspid atresia [6]. Although CHD affects both males and females equally, some lesions such as COA, AS, transposition of great vessels, and TOF show a male preponderance whereas females report more ASD [7].

The etiology of the majority of CHDs is not known. It is multifactorial and a combination of genetic predisposition and environmental stimulus play a major role [7]. CHD clinically presents according to the type and severity of the defect [8]. Among newborns, the common symptoms of CHD include fast breathing, bluish discoloration of the skin and mucosa, heart failure, and shock. On clinical evaluation, heart murmur and absent femoral pulse are common findings [8]. Infants and children present with breathlessness, clubbing, cyanosis, murmur, syncope, history of squatting, heart failure, rhythm disorder, and failure to gain weight [9]. CHD is a common congenital problem leading to psychological stress and financial burden to the family and increases morbidity and mortality. However, if the issue is identified at an initial age, the outcome is good and the survival rate is improved. With advancements in medical and surgical management, more children with CHD are getting cured and reaching adulthood [10].

Our hospital is a tertiary care center and caters to patients from Bihar, Jharkhand, West Bengal, Nepal, and other nearby areas. It has a well-established pediatric cardiology department. Bihar is India’s second-most populous state and is not very developed in many human parameters. Hence, a retrospective, cross-sectional study was conducted to determine the clinical and echocardiographic profile of CHDs in patients aged 0-12 years in a tertiary care medical institute in eastern India among the admitted children and outpatient department (OPD) patients.

## Materials and methods

Study design and participants

After obtaining ethical approval from the Institutional Ethics Committee of Indira Gandhi Institute of Medical Sciences (approval number: 339/IEC/IGIMS/2021), a retrospective, cross-sectional study was performed in the Pediatrics Department of Indira Gandhi Institute of Medical Sciences, Patna, Bihar from January 2019 to January 2021. This study aimed to determine the clinical and echocardiography profile of CHD among admitted children and outpatient department (OPD) patients. A total of 200 patients aged 0-12 were enrolled in this study.

The study design was exploratory, with a proforma drafted to investigate patients with features suggestive of CHD. During data collection, we noted that from January 2019, all pediatric echocardiography was performed by a trained cardiologist. Records were obtained from the departmental record-keeping register, and reports were available for analyzing the data.

Inclusion criteria included features suggestive of CHD in children aged 0-12 years. Patients aged greater than 12 years, with features not suggestive of CHD, and with innocent murmurs (functional) were excluded.

According to the objectives of the study, clinical data and clinical echocardiography correlation data were collected. History and physical examination provided a provisional clinical impression, and, subsequently, patients were subjected to routine tests, electrocardiograms, and roentgenographic studies.

The echo machine used was a Sonosite color Doppler cum echocardiography machine using probe P 10x for neonates and P 21x for pediatric cases. Review echocardiography was performed by a trained cardiologist using Philips EPIQ 7C, and the probes used were S 12-4 for neonates and S 8-3 for pediatric cases. Two-dimensional transthoracic echocardiography was obtained and an echocardiography diagnosis was derived in most cases. The clinical echocardiography correlation was obtained and the results were analyzed.

Statistical analysis

For data collection, cardiology and pediatric department registers were reviewed and all CHD data were collected. Data analysis was done using SPSS software version 25.0 (IBM Corp., Armonk, NY, USA). Categorical variables were measured as a percentage and standard deviation and mean for quantitative variables. Fisher’s exact test was used to infer any differences between categorical variables. P-values of less than 0.05 were considered statistically significant.

## Results

In total, 200 children with CHD were evaluated from January 2019 to January 2021 with 130 males and 70 females. Overall, 90 (45%) children were below one year of age. In total, 142 children were diagnosed with ACHD, while 58 children had CCHD. Out of the 200 cases of CHD, 110 belonged to the rural community and 90 to the urban community. Overall, 60% of all cases followed the Hindu religion and the rest were Muslim (Table 1).

**Table 1:** Characteristics of patients. ACHD: acyanotic congenital heart disease; CCHD: cyanotic congenital heart disease

Variable	Indices	Number (N = 200)	Percentage (%)
Sex	Male	130	65
	Female	70	35
Religion	Hindu	120	60
	Muslim	80	40
Area	Urban	90	45
	Rural	110	55
Type of lesion	ACHD	142	71
	CCHD	58	29

Among 200 cases of CHD, VSD constituted 62 cases accounting for 31% of all CHD cases, and 44% of all cases of ACHD belonged to VSD. ASD was the second most common CHD accounting for 23% of all CHD cases. TOF constituted 23 cases accounting for 11.5% of all cases. Of all CHD cases, CCHD was the most common pathology (39.6%). TGA and tricuspid accounted for 8% and 7%, respectively, of all CHDs. One case of interrupted aortic arch and one case of truncus arteriouses were also noted (Table 2).

**Table 2:** Echocardiographic profile of congenital heart diseases.

Type of congenital heart disease	Number (%) (N = 200)
Ventricular septal defect	62 (31)
Atrial septal defect	46 (23)
Patent ductus arteriosus	22 (11)
Tetralogy of Fallot	23 (11.5)
Coarctation of the aorta	2 (1)
Transposition of great arteries	8 (4)
Double outlet right ventricle	4 (2)
Tricuspid atresia	7 (3.5)
Total anomalous pulmonary venous return	2 (1)
Aortic stenosis	2 (1)
Single ventricle	4 (2)
Common atrium	3 (1.5)
Aortic stenosis + Coarctation of the aorta	2 (1)
Ventricular septal defect + Atrial septal defect	6 (3)
Truncus arteriosus	1 (0.5)
Ebstein anomaly	5 (2.5)
Interrupted aortic arch	1 (0.5)

Based on the age at the time of presentation, 90 (45%) cases were diagnosed below one year of age, 70 (35%) were diagnosed between one and five years of age, and the remaining 40 (20%) were diagnosed after five years of age (Table 3).

**Table 3:** Type of congenital heart diseases by age group. ACHD: acyanotic congenital heart disease; CCHD: cyanotic congenital heart disease; CHD: congenital heart disease

Age group	ACHD	CCHD	Total (CHD)
Below 1 year	59	31	90
1–5 years	52	18	70
Above 5 years	31	9	40
Total	142	58	200

Analyzing the symptoms of different CHDs, both ACHDs and CCHDs, the common symptoms were fast breathing, cough, feeding difficulties, and failure to thrive. The major signs of ACHD were chest retractions, fever, and features of CHF. CCHD presented as cyanosis, cyanotic spell, edema, and chest pain. CHF was most common in ACHD which was 30% compared to CCHD in which only 7% of cases fell under CHF (Table 4).

**Table 4:** Clinical presentation of congenital heart diseases. ACHD: acyanotic congenital heart disease; CCHD: cyanotic congenital heart disease; CHD: congenital heart disease

Mode of presentation	ACHD (%) (n = 142)	CCHD (%) (n = 58)	Total (%) (N = 200)
Fast breathing	51 (35)	25 (43)	76 (38)
Retraction of chest	56 (39)	34 (58)	90 (45)
Cough	46 (32)	31 (53)	77 (38)
Chest pain	17 (12)	6 (10)	23 (11.5)
Cyanosis	0	47 (81)	47 (23.5)
Cyanotic spell	0	18 (31)	18 (9)
Difficulty during feeding	39 (27)	29 (50)	68 (34)
Edema	9 (6)	2 (3)	11 (55)
Fever	62 (43)	26 (44)	88 (44)
Failure to thrive	74 (52)	36 (62)	110 (55)
Congestive heart failure	42 (30)	4 (7)	26 (13)

## Discussion

This is the first study exploring the clinical spectrum and prevalence of CHDs in the Bihar region in the pediatric age group. In our study, out of a total of 200 cases, ACHDs constituted 142 cases accounting for 71% of total CHDs, which is similar to other studies [11-14]. CCHDs constituted 58 cases accounting for 29% of all CHD cases, which is similar to previously reported studies [15-18]. In our study, the male-to-female ratio was 1.8, which is similar to the study by Hussain and Kumar et al. [10,12]. Overall, 45% of CHD cases presented at less than one year of age, which is similar to the study by Abqari et al. [18]. The most common CHDs diagnosed were VSD (31%), which is similar to reported cases in other countries [19,20]. ASD was the second most common CHD (23%), which is similar to a study by Xuan et al. [21]. It was also the most common form of CHD which is frequently missed during childhood. The most common CCHD lesion was TOF (11.5%), which is similar to other previously reported studies [22,23]. Definitive diagnostic features of TOF and other associated cardiac abnormalities were identified by echocardiography. A pulse oximeter is an important tool in the evaluation of patients with congenital heart disease, especially in cyanotic congenital heart diseases. 90(45%) children with CHDs were below one year of age. The increased frequency of CHDs in this age group could be explained by the fact that more cases are discovered antenatally to have suspected CHD which helps in avoiding delayed diagnosis and keeping down the related outcomes [24,25]. On observing the clinical features of CHD, fast breathing, poor weight gain, cough, feeding difficulties, palpitation, cyanosis, and edema were the major features, and this observation correlated well with other studies conducted in different countries [1,26]. In our study, 30% of CHD patients presented with features of CHF, which was statistically significant. Similarly, Sommers et al. [27] and Al Faham et al. [28] detected heart failure in 39.1% and 44% of patients, respectively, with CHD. Increased prevalence of heart failure in ACHD is primarily viewed as a result of a volume or pressure overload.

Study limitations

This study had some limitations. The clinical and echocardiographic profile of CHDs described in this study among children was mainly referred from another center because our hospital is the only superspecialty hospital in Bihar. Hence, patients with asymptomatic or milder lesions such as ASDs were likely under-represented. Further, this work was not designed to study the risk factors as predictors for the occurrence of CHDs.

## Conclusions

This retrospective, cross-sectional study analyzed the spectrum and clinical presentations of CHDs in children in a tertiary health facility in Bihar. Among ACHD patients, 31% VSD and 21% ASD cases were noted. In CCHD, TOF was the most common with 11.5% of cases. One case of interrupted aortic arch and truncus arteriosus was also noted. Respiratory tract conditions were the most common comorbidities encountered. This is one of the first studies in the Bihar region in the pediatric age group and can help to know the prevalence of CHDs in this region. Further, it can help the stakeholders to devise policies for better management of CHDs.
